# Short-Term Estrogen Replacement Effects on Insulin Sensitivity and Glucose Tolerance in At-Risk Cats for Feline Diabetes Mellitus

**DOI:** 10.1371/journal.pone.0130696

**Published:** 2015-06-18

**Authors:** Allison Wara, Sara Hunsucker, Krystal Bove, Robert Backus

**Affiliations:** Department of Veterinary Medicine and Surgery, University of Missouri, College of Veterinary Medicine, Columbia, Missouri, United States of America; Shanghai University of Traditional Chinese Medicine, CHINA

## Abstract

Male domestic cats that are neutered and overweight are at an increased risk for developing a type-2-like diabetes mellitus. Beneficial effects of 17β-estradiol (E2) on glucose homeostasis may be lost with neutering and thereby account for increased diabetes risk. To evaluate this, adult male neutered overweight cats (n=6) were given daily E2 (1.0 μg/kg) or vehicle (Vh; ethanol, 1.0μL/kg) in a single crossover trial of 14-day periods with a 7-day washout. The E2 and Vh were voluntarily ingested on food. The E2 dosage was determined in a pre-trial to significantly and transiently reduce food intake with no measurable change in plasma E2 concentration. During treatments, physical activity was assessed with collar-mounted accelerometers on days 9-11, and tests of intravenous insulin tolerance and intravenous glucose tolerance were conducted on days 13 and 14, respectively. Over the 14 days, E2 compared to Vh treatment reduced (p=0.03) food intake (- 22%) but not enough to significantly reduce body weight; activity counts were not significantly changed. With E2 compared to Vh treatment, the late-phase plasma insulin response of the glucose tolerance test was less (p=0.03) by 31%, while glucose tolerance and insulin sensitivity indexes were not significantly changed. The results indicate that oral E2 at a dosage that moderately affects food intake may reduce insulin requirement for achieving glucose homeostasis in neutered male cats. Further investigation is needed to identify the mechanism underlying the E2 effect.

## Introduction

In North America, obesity is the most common form of malnutrition in dogs and cats [1]. It is estimated that 35.1% of cats over one year of age are either overweight or obese [2]. Risk factors for feline obesity include gender (males > females), reproductive status (neutered > intact), indoor confinement, and physical inactivity [3]. To date, the most commonly implicated risk factor is gonadectomy which is the single most important factor in cats [4]. Obesity appears to predispose this species to a variety of diseases including diabetes mellitus (DM), specifically in overweight neutered males [2].

Post-gonadectomy weight gain has been previously attributed to loss of inhibition of food intake by estrogens in both male and female cats [5]. It has been demonstrated that supplementation of a physiologically relevant dose of 17β-estradiol (E2) to gonadectomized cats results in a reduction in hyperphagia [6] and is effective in the prevention of post-gonadectomy weight gain [7]. These studies uncovered the key role of gonadal E2 in the modulation of food intake in cats.

Substantial evidence also demonstrates that E2 has antidiabetic properties in humans [8], [9] and rodents [10], [11] when ovarian production ceases, or is absent, by enhancing insulin sensitivity and β-cell function. Postmenopausal women are at risk of developing visceral obesity, insulin resistance, and type 2 DM while ovariectomized rodents are more susceptible to hyperglycemia. In both of these models, symptoms can be reversed with exogenous E2 therapy.

It is also observed in rodent models that gonadectomy significantly reduces physical activity in both males and females and that supplementation with E2 recovers pre-neutering conditions [12]. Since physical inactivity is associated with feline DM [3] and increased activity is recognized as a contributing factor in the prevention of type 2 DM in humans [13], it would be interesting to determine if E2 supplementation restores physical activity in cats back to pre-neuter conditions, particularly in those at increased risk of DM (obese neutered males).

Although ample data supports a beneficial role of E2 on glucose homeostasis and physical activity in various species, there is a paucity of information available in domestic cats. To our knowledge, no study to date has examined the impact of exogenous E2 on indices of glucose homeostasis or physical activity in at-risk cats. In the present study, we hypothesized that oral replacement of gonadal estrogen lost with neutering would increase insulin sensitivity and glucose tolerance and restore physical activity to pre-neuter conditions. Findings in support of this hypothesis may indicate a benefit for E2 in the management and prevention of feline diabetes, a condition of insulin resistance and diminished insulin secretion [14].

## Materials and Methods

### Ethics Statement

Approval for the study was acquired from the University of Missouri Animal Care and Use Committee (ACUC). No animals used in the study were sacrificed; they were retained at the University for subsequent nutrition research.

### Animals

Eight adult male neutered domestic shorthair cats were obtained from a vendor (Liberty Research Inc., Waverly, NY, USA) or purpose-bred at the University. Cats were variably overweight as a result of *ad-libitum* feeding with body condition scores of 5–9 on a 9-point scale (where 5/9 is ideal, 1/9 is emaciated, and 9/9 is severely obese [15]) but were otherwise healthy as determined from pre-trial physical exam, complete blood cell count, and serum biochemistry findings. Cats were housed in individual cubicles (1.1 x 1.6 x 2.4 m) overnight (12 hours) and were allowed to interact with each other in an adjacent central room during the day (12 hours). The room was temperature (23–27°C) and light (12:12 h light: dark) controlled.

### Diet

A commercially available, complete and balanced, dry, feline maintenance diet (Purina Pro Plan Urinary Tract Health Formula, Nestlé Purina PetCare Company, St. Louis, MO, USA) was offered to cats for *ad libitum* consumption (Table 1). The diet reflected a typical maintenance formula for indoor cats and supplied 18.3 kJ/g of metabolizable energy (ME). The distribution of protein, fat, and carbohydrates on an ME basis was estimated to be 34.1, 23.5, and 42.3% respectively.

**Table 1:** Proximate analysis and ingredient list of the diet^a^.

Nutrient	g/kg
Crude protein	33.8
Crude fat	9.6
Crude fiber	1.1
Moisture	8.3
Ash	53

Ingredients^b^: Corn gluten meal, chicken, wheat flour, brewers rice, ground yellow corn, animal fat preserved with mixed-tocopherols, egg product, sodium caseinate, phosphoric acid, calcium carbonate, potassium chloride, animal digest, salt, L-Lysine monohydrochloride, dried whey, choline chloride, dicalcium phosphate, taurine, zinc sulfate, ferrous sulfate, manganese sulfate, Vitamin E supplement, niacin, citric acid, Vitamin A supplement, calcium pantothenate, thiamine mononitrate, copper sulfate, riboflavin supplement, Vitamin B-12 supplement, pyridoxine hydrochloride, folic acid, Vitamin D-3 supplement, calcium iodate, biotin, menadione sodium bisulfite complex, sodium selenite.

^a^As determined by the University of Missouri Agricultural Experiment Station Chemical Laboratories.

^b^As reported on the product label.

### Study Design

Prior to experimentation, a dose response trail was conducted to select an appropriate dosage of E2 for subsequent evaluation of glucose tolerance and insulin sensitivity. Eight adult male neutered cats [2.3–6.1 (median = 5.8) years] were randomly assigned to four groups of 2 cats each. Cats ranged in body weight from 4.44–8.48 (median = 7.1) kg and were 1.5–4.5 (median = 4.5) years post-neutering. For four successive periods of 14 days, three dosages of E2 (0.5, 1.0, 2.0 μg/kg) and vehicle (Vh, 1.0 μL/kg of ethanol) were given to each cat in a Latin-squared design with no intervening periods of washout. During the first 5 days of each treatment period, the cats voluntarily ingested E2 or Vh applied to a food kibble immediately before diet presentation. Food intake was determined daily. On day 6 of treatment periods, prior to diet presentation, approximately 14 hours following ingestion of E2 or Vh, jugular venous blood (6 mL) was collected by venipuncture and transferred to glass tubes containing potassium EDTA. Plasma extracted from the blood was stored at -20°C until later E2 concentration determination.

For evaluation of E2 on glucose tolerance, insulin sensitivity, and physical activity, a crossover trial of 5 weeks in duration was conducted on 6 adult [3.1–6.8 (median = 6.5) years] male neutered cats [2.0–5.2 (median = 5.2) years post-neuter] that were overweight [5.17–8.28 (median = 7.13) kg] with body condition scores of 6-9/9 on a 9-point scale [15]. Prior to evaluation, body fat percentages of all cats were estimated by measurement of labeled water (deuterium oxide, ^2^H_2_0) enrichment in serum collected from jugular blood, 3 hours after subcutaneous injections of ^2^H_2_0 (0.4 mL/kg) in 0.9% sterile-filtered saline as previously described [16].

**Fig 1:**
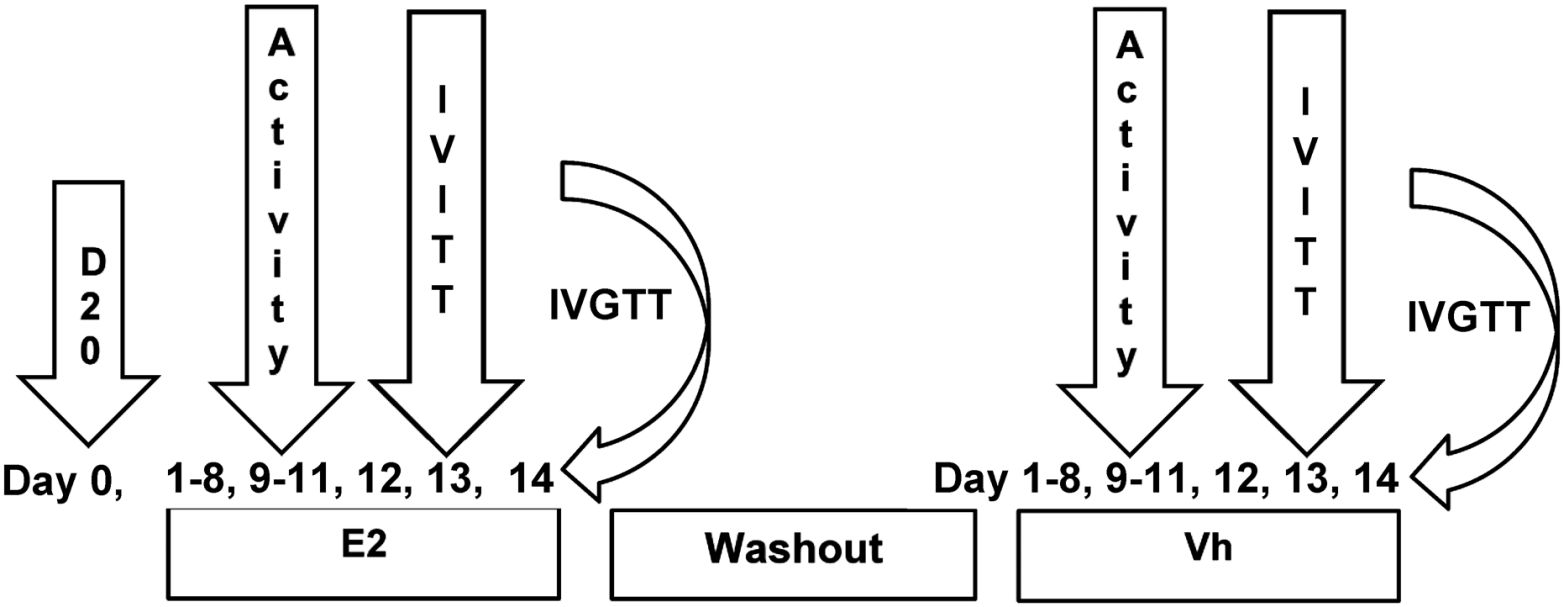
Experimental protocol. Figure of experimental protocol of one of two groups of the single crossover trial to illustrate the order of events. The treatment order of the second group was reversed. D20, deuterium oxide dilution analysis; IVITT, intravenous insulin tolerance test; IVGTT, intravenous glucose tolerance test; Vh, vehicle; E2, estradiol.

The cats were randomly assigned into 2 groups of 3 according to treatment. Treatments consisted of E2 at a daily dose of 1.0 μg/kg or vehicle on food as described for the dose response trial. Treatments were administered for 14 consecutive days prior to overnight meal presentation. Following a 1-week washout period, the treatments were repeated but crossed-over such that after 5 weeks, each subject received both E2 and Vh (Fig 1). A two-week treatment period was used such that changes in variable observations were attributable to treatment, not an effect of substantial weight change.

### Activity Monitoring

On days 9–11 of each treatment period, Actical Activity Monitoring Devices (Mini Mitter, Bend, OR, USA) were affixed to pet collars to evaluate physical activity (Fig 1). The monitors were previously validated for measurement of physical activity in cats [17].

Activity counts were logged at 15 second intervals and the average number of activity counts/day was determined for each cat over 72 hours of continuous recording.

### Glucose and Insulin Tolerance Tests

On day 12 of each treatment block, cats were sedated with subcutaneous injections of acetylpromazine (0.1 mg/kg), atropine sulfate (0.04 mg/kg), and butorphanol (0.2 mg/kg). During sedation, cephalic catheters were placed (22 ga., 2.5cm; BD Insyte, Sandy, UT, USA) followed by administration of propofol (4 mg/kg) and implantation of jugular venous catheters (18 ga.,12 cm; Cook Medical, Bloomington, IN, USA). The jugular and cephalic catheters were bandaged and maintained patent with flushing of an anticoagulant solution (0.38% sodium citrate in 0.9% sodium chloride) every 8 hours until removal on day 14.

On day 13 of each treatment block, an intravenous insulin tolerance test (IVITT) was performed on each cat following food withholding of 12 hours (Fig 1). A first blood sample of 1 mL was drawn from jugular catheters to establish baseline glycemia levels, followed by a 0.1 unit/kg infusion of insulin (Humilin R, Eli Lilly, Indianapolis, IN, USA) in cephalic catheters. The insulin dosage is a standard used for IVITT across species–cats [18], [16], humans [19], [20], dogs [21], [22], pigs [23], and calves [24]. Fifteen minutes after infusion, a second blood sample of 1 mL was drawn from jugular catheters. Blood samples were transferred to glass anticoagulant tubes (4.5 mg K_3_ EDTA, BD Vacutainer, Franklin Lakes, NJ, USA) and stored on ice for transport. All samples were centrifuged and plasma was stored at -20°C for assays. An insulin sensitivity index (*k*_ivitt_) was calculated using the equation: *k*_ivitt_ = ([glucose]_baseline_-[glucose]_15min_)/[glucose]_baseline_.

On day 14 of each treatment block, an intravenous glucose tolerance test (IVGTT) was performed on each cat following food withholding of 12 hours (Fig 1). Two mL of blood were drawn from jugular catheters immediately before and at 5, 10, 15, 30, 45, 60, 90, and 120 minutes after infusion of 1.0 g/kg of dextrose solution (50 g/dL, Vedco Inc., Saint Joseph, MO, USA) in cephalic catheters over 30–45 s. The dextrose dose has previously been shown to reveal glucose tolerance and insulin sensitivity differences in lean and obese cats [25]. Blood samples were transferred to glass anticoagulant tubes (4.5 mg K_3_EDTA, BD Vacutainer, Franklin Lakes, NJ, USA) and stored on ice for transport. Samples were processed and stored as described for the IVITT procedure. Previously described variables of glucose tolerance and insulin sensitivity (linear phase of rate of glucose disappearance, AUC_0–120_, plasma insulin concentration at time 0, late phase insulin AUC, insulin sensitivity index, and insulin glucose ratios) were determined [26].

Glucose disappearance was evaluated in terms of half-time of the rate of decline in plasma glucose between 15–90 min after dextrose injection of the IVGTT. The timing of measurement of the slope is in accord with precedent, in research that demonstrates reduced glucose tolerance in feline obesity [26]. However, a longer interval for slope calculation (75 vs. 30 min) was used because the apparent linear-phase of the decline in plasma glucose during the IVGTT extends to 90 min.

### Laboratory Analyses

Plasma E2 concentration was determined as previously described [27] with a minor modification: In place of tritiated E2, estrone was added as internal standard to plasma samples (1 mL) prior to solid-phase extraction. The estrone in HPLC fractions of plasma extracts were quantified with the same commercially available assay, an RIA used for E2 quantitation (ImmuChem Double Antibody, MP Biomedicals, Cosa Mesa, CA). Estrone was a suitable internal standard because the RIA was substantially cross-reactive with the estrone while endogenous estrone in cat plasma is very low (~30 pg/mL, [27]) compared to the amount added as internal standard (1000 pg). The concentration of estrone that displaced 50% of the RIA radio-ligand (17β-estradiol-^125^I) was approximately 20-times that of E2.

A commercially available radioimmunoassay kit was used to measure plasma insulin concentrations (PI-12K, EMD Millipore Corporation, Billerica, MA, USA). This method was previously validated for use in cat plasma [16]. Plasma glucose concentrations were determined using the manual method of a commercially available colorimetric assay based on glucose oxidase (Sigma-Aldrich, St. Louis, MO, USA).

### Statistical Analyses

All statistical analyses were performed using the SAS 9.3 software package (SAS Institute Inc., Cary, NC, USA). Differences between Vh and E2 treatments were compared using the Wilcoxon signed-rank test for paired observations and the Mann-Whitney test for independent observations. Spearman rank correlation analysis was used to determine the significance of associations between body weight or fat with changes in food intake, activity level, *k*_ivitt_, and IVGTT late-phase insulin response. A p-value ≤ 0.05 was considered significant. All values of pooled observations are reported as medians. Variance in observations about medians are indicated in reported in range and/or 25^th^ and 75^th^ percentile values. The AUC for plasma glucose and insulin concentrations was determined by the trapezoidal method [28].

## Results

### E2 Dose Response Trial

**Fig 2:**
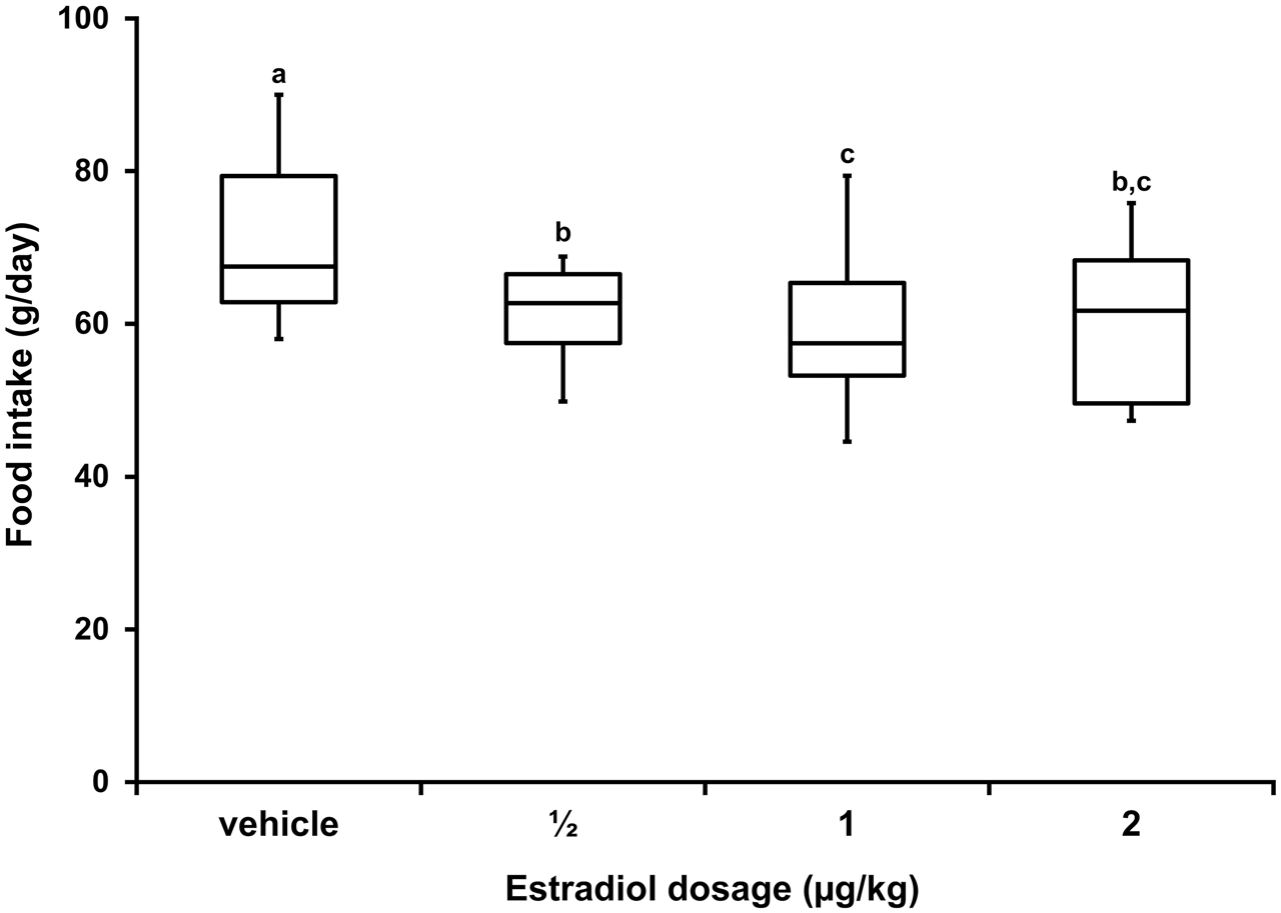
Box-whisker plots demonstrating results of food intake over successive 3-day intervals prior to and during 14-day treatment periods when vehicle, 0.5, 1.0, or 2.0 μg/kg of E2 were ingested daily for 5 days. Boxes represent the 25 to 75^th^ percentile, central lines represent medians, and whiskers show the minimum and maximum observations on 8 cats. Where letters above plots are different, food intake observations are different (p≤0.05).

A mean of daily food intake was determined for each cat over the 5 days during which E2 or vehicle was ingested. The mean daily food intakes associated with all E2 dosages were less (p<0.04) than those associated with vehicle ingestion. The mean daily food intakes when E2 dosage was 1.0 μg/kg were less (p = 0.05) than those when the E2 dosage was 0.5 μg/kg, and not different (p = 0.84) from those when the E2 dosage was 2.0 μg/kg (Fig 2).

**Fig 3:**
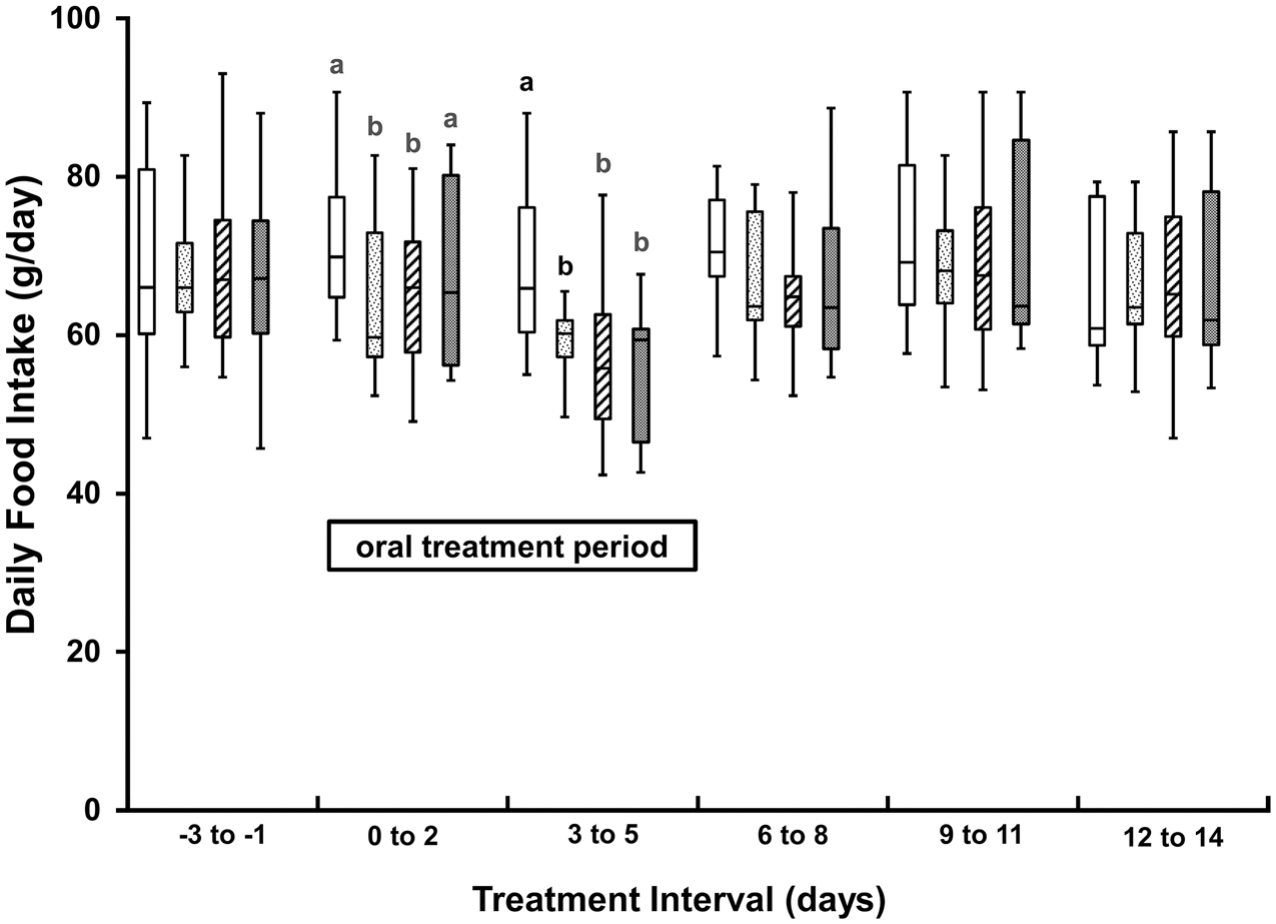
Box-whisker plots of daily food intake of 8 cats during 3-day periods preceding (days -3 to -1), during (days 0 to 5), and after (days 6 to 14) oral ingestion of vehicle (open boxes) and E2 at dosages of 0.5 (stippled boxes), 1.0 (diagonal line boxes), and 2.0 (shaded boxes) μg/kg body weight. Boxes represent the 25 to 75^th^ percentile, central lines represent medians, and whiskers show the minimum and maximum observations on 6 cats. Food intake observations within 3-day periods are different (p≤0.05) where letters above plots are different.

The plasma E2 concentrations observed after 0.5, 1.0, and 2.0 μg/kg E2 treatments (median [range]): 4.8 [6.8–54.3], 4.4 [1.6–29.9], and 3.6 [1.1–36.7] pg/mL, respectively) were not significantly different from those after vehicle treatments (3.0 [1.3–39.9], pg/mL). The E2 effect on food intake soon waned following the withdrawal of E2. Mean daily food intakes were determined over successive 3-day intervals during the vehicle and E2 treatment periods. The mean daily food intakes of days 3 to 5 of all E2 treatment periods were less (p<0.04) than the same intervals of the vehicle treatment periods (Fig 3). The mean daily food intakes of days 0 to 2 were also significantly lower for the 0.5 and 1.0 μg/kg E2 treatment periods (p<0.01), but not for the 2.0 μg/kg E2 treatment periods (p = 0.15). The mean daily food intakes of days 6 to 8, 9 to 11, and 12 to 14 were not significantly different between vehicle and E2 treatment periods.

### Effects of E2 on Body Composition and Food Intake

**Fig 4:**
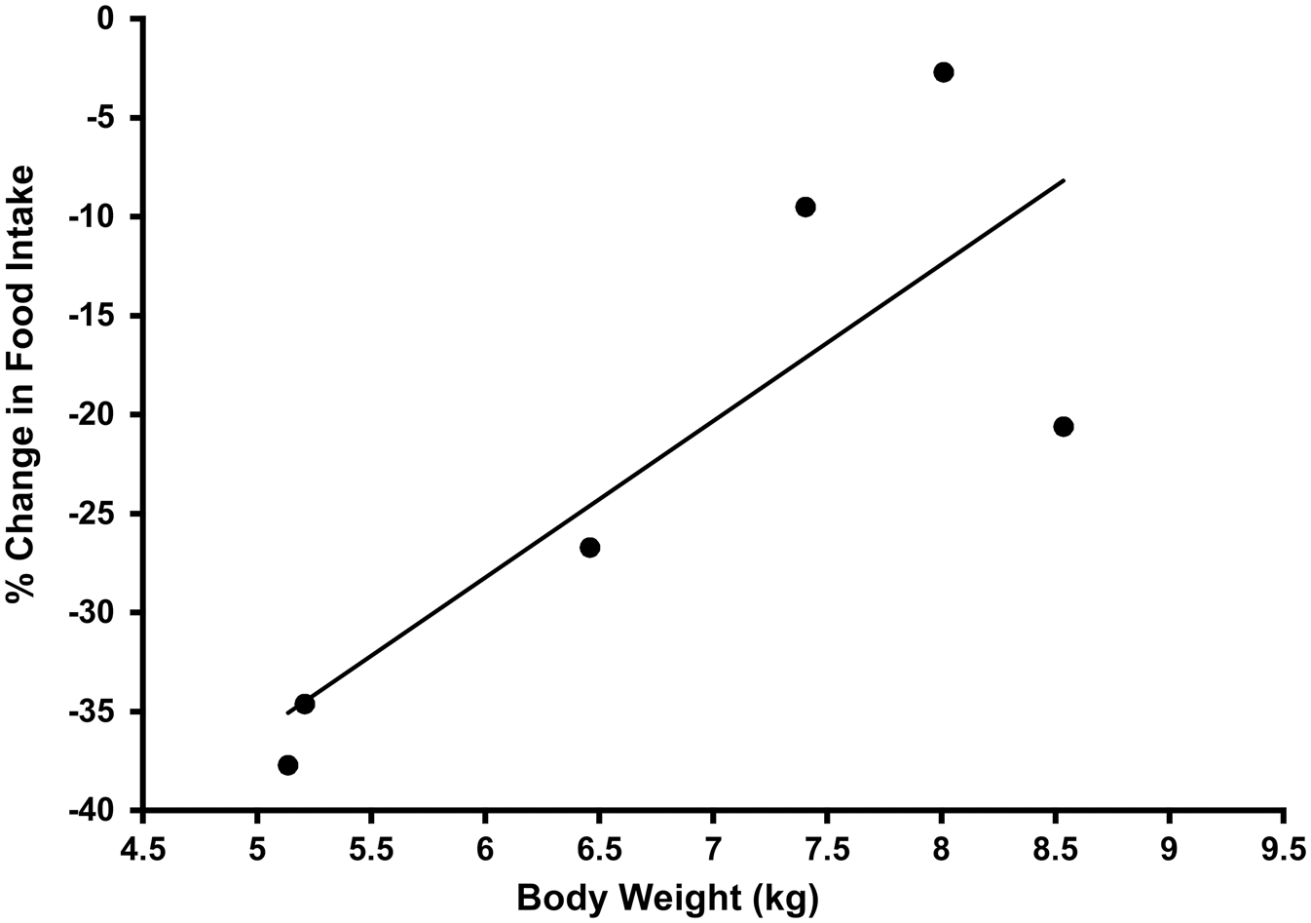
Correlation between body weight (kg) and % change in food intake during E2 treatment (*ρ* = 0.83 [p = 0.04]).

Body fat content among the cats varied substantially and was greater than ideal (> 25% of body weight) (Table 2). For each cat, mean daily food intake during the first 11 days of each treatment period was determined. Food intake observations from days 12 to 14 were excluded from analyses because of scheduled food withholding needed for catheter placement and tests of insulin and glucose tolerance. The food intake means were pooled by treatment period for median and variance determinations and evaluation of treatment effects. The E2 treatment significantly (p = 0.03) reduced food intake by a median of 22%/day compared to Vh (Table 3). Despite decreased food consumption, no significant change was observed in body weight with E2 treatment. The percentage decrease in food intake caused by E2 treatment (a measure of effectiveness of E2 in reducing food intake) was calculated for each cat. The potency of the E2 treatment on food intake varied among the cats. Correlation analysis revealed an association between cat body weight and the effectiveness of E2 in reducing food intake (p = 0.04, ρ = 0.83) (Fig 4). This relationship indicated that the leaner cats had a significantly greater decrease in food intake in response to E2 treatment compared to cats that were more obese.

**Table 2:** Median, minimum (min) and maximum (max) age, body weight (kg), lean body mass (kg), fat mass (kg) and fat mass (%) as determined by deuterium oxide dilution analysis prior to experimental trials.

	Median	Min	Max
Age (y)	6.5	3.1	6.8
Body weight (kg)	7.13	5.17	8.28
Lean mass (kg)	3.38	3.08	5.36
Fat mass (kg)	3.42	1.79	4.16
Fat mass (%)	45.5	34.7	56.4

**Table 3:** Effect of oral estradiol treatment (E2) (1.0 μg/kg) on body weight, food intake and energy intake.

	Vh median (range)	E2 median (range)
Body weight Day 1 (kg)	6.89 (5.14–8.23)	7.15 (5.04–8.47)
Body weight Day 11^b^ (kg)	6.93 (5.14–8.54)	6.95 (4.98–8.47)
Change in body weight (%)	1.30 (-0.60–3.70)	-1.10 (-2.8-[-0.10])
Food intake (g/day)^c^	66.4 (57.8–83.5)	51.6 (42.7–66.3)^a^
Food intake(g/kg/day)^c^	9.4 (7.8–15.9)	8.0 (6.6–10.2)^a^
Energy intake (KJ/kg/day)	172 (142–290)	146 (120–186)^a^

^a^Significantly (p = 0.03) different from the corresponding vehicle value.

^b^Body weights between days 12–14 were not recorded due to necessary food withholding.

^c^Average determined over 11 days. Food intake for days 12–14 were not recorded due to necessary food withholding for catheterization and tolerance testing.

### Effects of E2 on Physical Activity

The collar-mounted activity monitors were well tolerated. The two housing types (individual cubicles vs central room for group interaction) created a wide variation in movements, with peak activity occurring during periods of group interaction. The range in daily activity counts relative to the calculated means varied greatly within cats. Overall, the effect of E2 on physical activity was not significant (p = 0.19) (Table 4). The percentage change in activity counts with E2 treatment was not significantly correlated with body fat (p = 0.21). However, the 3 cats with the lowest body fat (28–37%) had a greater (p = 0.05) increase in physical activity with E2 treatment compared to that of cats with the greatest body fat (51–56%).

**Table 4:** Effects of oral estradiol (E2) (1.0 μg/kg) and control vehicle (Vh) (1.0μL/kg) on study outcomes in overweight adult male cats (median [range], n = 6).

**Intravenous glucose tolerance test (IVGTT)**	**Vh**	**E2**	**p =**
Plasma glucose *t*_0_ (mmol/L)	4.7 (3.8–6.1)	5.4 (3.7–6.9)	0.69
*K*_15–90_ (%/min)	-1.94 (-2.35-[-1.11])	-1.38 (-2.62-[-0.70])	0.22
Glucose AUC_0–120_ (mmol/L · min)	1920 (1650–2740)	2220 (1610–2630)	0.12
Insulin *t*_0_ (μU/mL)	14 (9–27)	15 (9–22)	0.84
Insulin AUC_0–15_ (μU/mL · min)	401 (202–483)	285 (178–484)	0.44
Insulin AUC_60–120_(μU/mL · min)	4630 (1300–5290)	3190 (1160–4820)	0.03^a^
**Intravenous insulin tolerance test (IVITT)**	**Vh**	**E2**	**p =**
Plasma glucose at baseline (mmol/L)	4.8 (3.7–5.0)	4.2 (3.4–4.8)	0.30
*k*_ivitt_	0.59 (0.43–0.75)	0.56 (0.30–0.77)	0.31
**Insulin/glucose ratio**	**Vh**	**E2**	**p =**
*t*_0_	0.144 (0.102–0.404)	0.168 (0.075–0.237)	1.00
AUC_0–15_	0.052 (0.024–0.069)	0.037 (0.023–0.058)	0.69
AUC_60–120_	0.414 (0.190–0.602)	0.249 (0.146–0.377)	0.03^a^
**Activity**	**Vh**	**E2**	**p =**
Activity counts/day	48270 (24993–67052)	51534 (38477–79115)	0.19

^a^Different from the mean of the corresponding variable.

### Effects of E2 on IVITT

Intravenous insulin tolerance tests were successfully conducted in all cats; hypoglycemic side effects were not observed. Correlations were determined between the k_*ivitt*_ of the IVITT during vehicle treatments and several other indirect indicators of insulin sensitivity that significantly correlate with the insulin sensitivity index of the Bergman Minimal Model, frequently-sampled IVGTT [29]. The k_*ivitt*_ values were significantly correlated (p < 0.05) with baseline plasma insulin concentration, the homeostatic model assessment (HOMA) index, the quantitative insulin check index (QUICKI), IVGTT plasma insulin concentration at 120 min, and the IVGTT AUC insulin concentrations between 60 and 120 min (Table 5).

**Table 5:** Effect of estradiol (1.0 μg/kg) on reputed insulin sensitivity indicators determined from intravenous glucose tolerance test (IVGTT) results (n = 6) and correlation of the indicators with the insulin sensitivity index (k_ivitt_) of the intravenous insulin tolerance test (IVITT).

	Difference from estradiol treatment^a^	Correlationwith k_ivitt_ ^b^	
Indicator^c^	p	ρ	p
IVGTT baseline plasma insulin	ns	-0.83	0.04
IVGTT baseline plasma insulin/glucose	ns	-0.71	ns
IVGTT 60 min plasma insulin	ns	-0.20	ns
IVGTT 120 min plasma insulin	ns	-0.94	0.01
IVGTT AUC_0–120_ min insulin	0.03	-0.60	ns
IVGTT AUC_60–120_ min insulin	0.03	-0.89	0.02
IVGTT HOMA	ns	-0.94	0.01
IVGTT QUICKI	ns	0.94	0.01
IVITT k_ivitt_	ns	-	-

^a^Significance (p =) of Wilcoxon signed-rank test of differences between vehicle and estradiol treatment observations on the indicator. ns = not significant, p > 0.05.

^b^Spearman coefficient (ρ) and significance (p =) of correlation between observations during vehicle treatment. ns = not significant, p > 0.05.

^c^AUC = area-under-the-curve. HOMA = (baseline insulin × baseline glucose)/22.5. QUICKI = 1/[log(baseline insulin) + log(baseline glucose)]. k_ivitt_ = (baseline plasma glucose - 15 min plasma glucose)/baseline plasma glucose.

Prior to insulin administration, baseline serum glucose concentrations were not significantly different between treatment groups (Table 4). At 15 minutes after infusion of insulin, glucose concentrations were not found to be statistically different with E2 treatment. The insulin sensitivity index, *k*_ivitt_, was not significantly changed by E2 (p = 0.31) (Table 4). With increasing body weight, *k*_ivitt_ decreased (ρ = -0.94, p<0.01) during E2 treatment but it was not significantly decreased during Vh treatment (ρ = -0.77, p = 0.07).

### Effects of E2 on IVGTT

**Fig 5:**
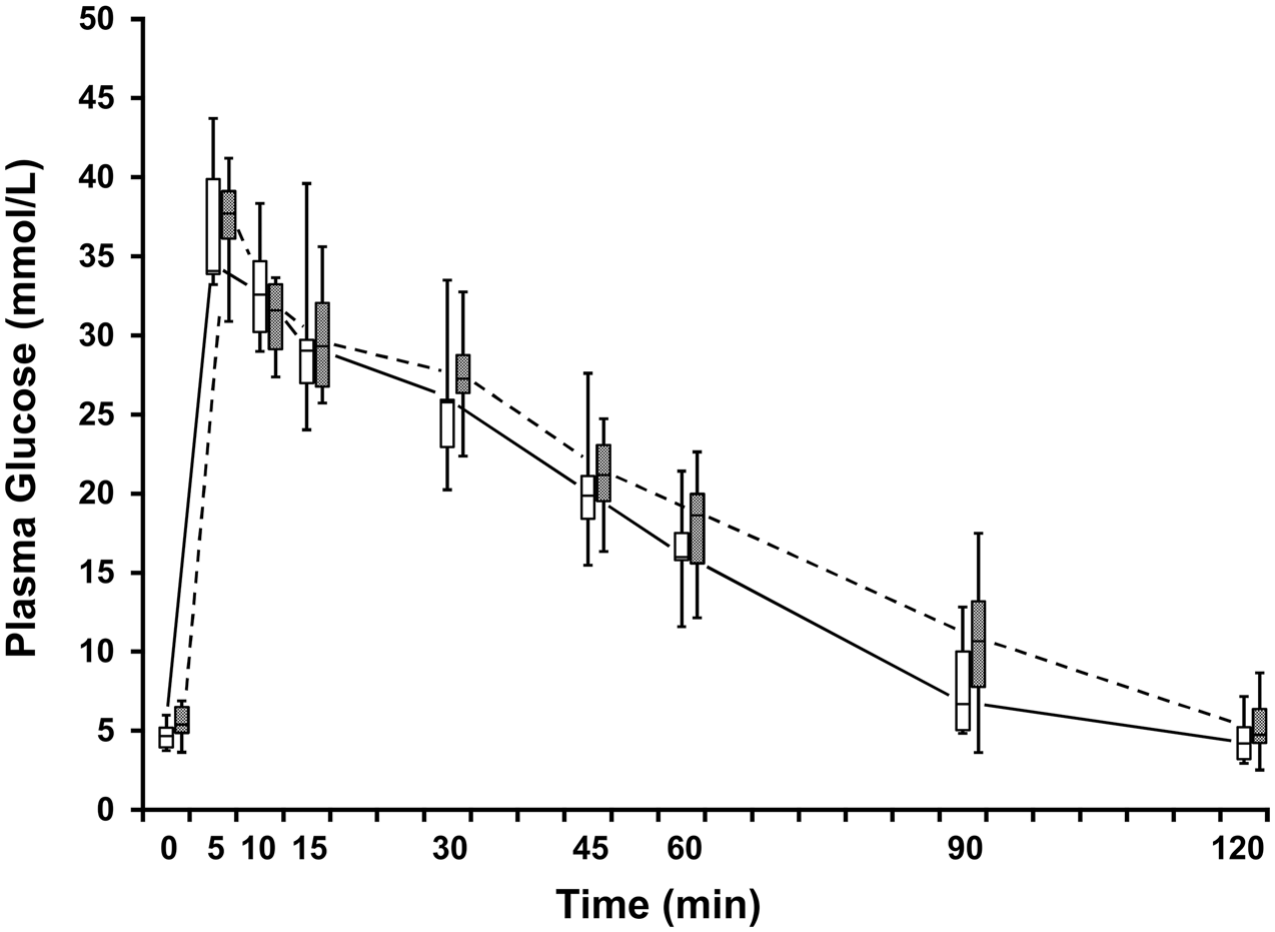
Box-whisker plots of plasma glucose concentrations (mmol/L) of the IVGTTs of adult, overweight (>25% body fat) male, neutered cats (n = 6) when given vehicle (open boxes) and 1.0 μg/kg E2 (shaded boxes). Boxes represent the 25 to 75^th^ percentile, central lines represent medians, and whiskers show minimum and maximum observations.

**Fig 6:**
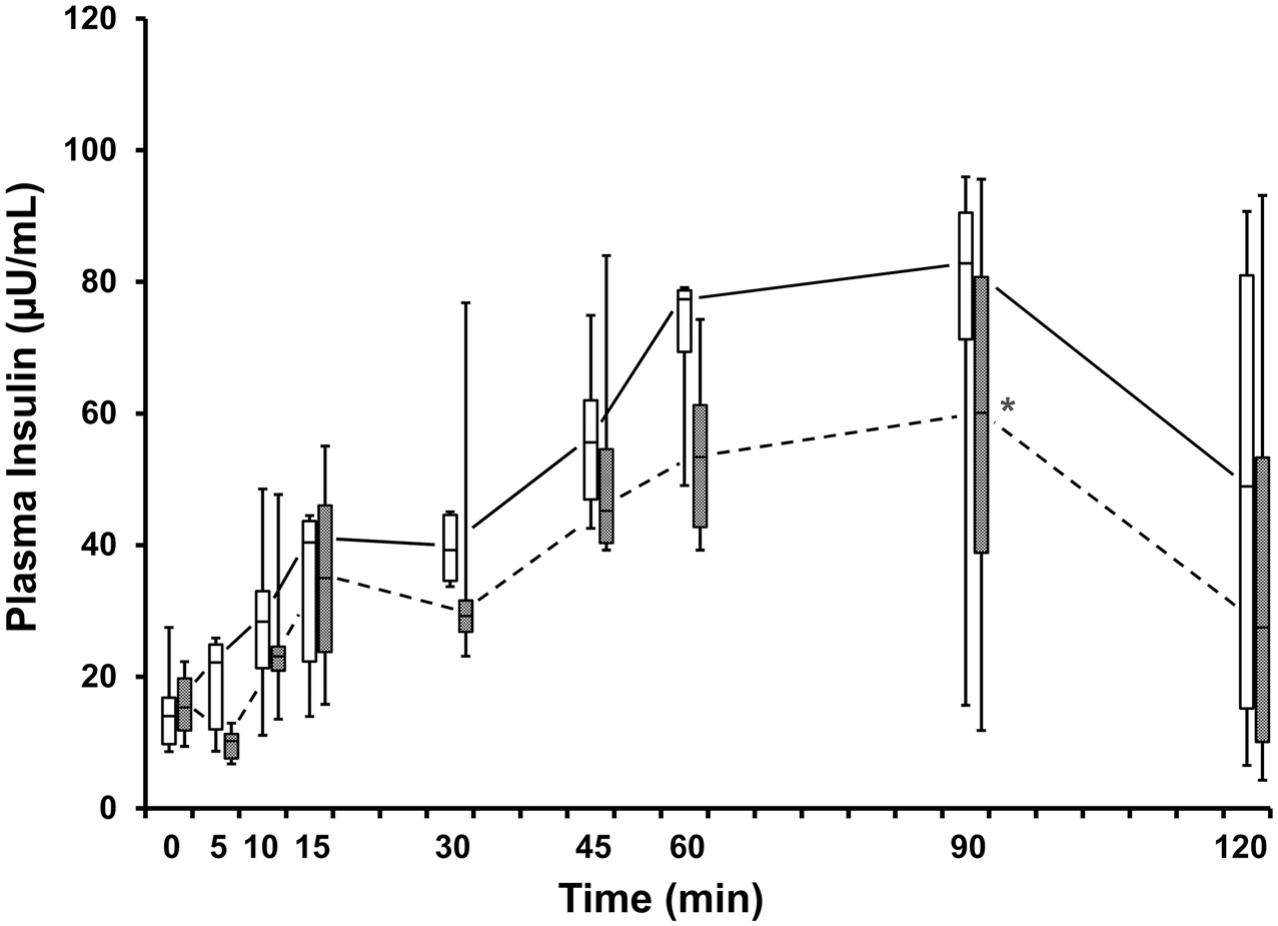
Box-whisker plots of plasma insulin concentrations (μU/mL) of the IVGTTs of adult, overweight (>25% body fat), male, neutered cats (n = 6) when given vehicle (open boxes) and 1.0 μg/kg E2 (shaded boxes). Boxes represent the 25 to 75^th^ percentile, central lines represent medians, and whiskers show minimum and maximum observations. Asterisk indicates difference (p = 0.03) between E2 and Vh treatment observations at 90 min.

Plasma glucose concentrations were not statistically different after E2 treatment at any sampling time of the IVGTT (Fig 5). The plasma insulin AUC concentration during the first 15 min, i. e., the early-phase insulin response (AUC _0–15 min_), did not significantly change with E2 treatment (Table 4). However, during the last hour, i. e., the late-phase insulin response (AUC _60–120 min_), plasma insulin concentration decreased (p = 0.03) (31%) with E2 treatment (Fig 6). With increasing body weight, the AUC _60–120 min_ increased (ρ = -0.89, p = 0.02) during Vh treatment. However, a significant relationship between body weight and AUC _60–120 min_ was not found during E2 treatment (p = 0.15).

The insulin to glucose ratio was less (p = 0.03) less with E2 compared to Vh treatment during the late-phase (insulin/glucose AUC _60–120 min_), but it was not found to be significantly different during the early-phase (insulin/glucose AUC _0–15 min_) or at baseline (insulin/glucose at *t*_0_) (Table 4).

## Discussion

### E2 Dose Response Trial

In the present study, we hypothesized that oral replacement of gonadal estrogen that is lost with neutering would result in improved glucose tolerance and insulin sensitivity and increased physical activity. From the results of the E2 dose response trial, the dosage of 1 μg/kg/day significantly reduced *ad libitum* food intake but did not significantly alter plasma E2 levels, indicating that the regimen was not supraphysiological but also not emulating of the pre-gonadectomy condition. Intact compared to neutered male cats have approximately twice as great plasma E2 concentrations [27]. A lacking effect of oral E2 on E2 in plasma collected several hours later is not surprising because E2 is rapidly conjugated in cats as in other species [30].

### E2 Effects on Food Intake

At 1.0 μg/kg, E2 decreased *ad libitum* food intake by 22% per day (Table 3). For reference, the dosage used was 10-fold greater than the subcutaneous dosage of E2 (~0.1 μg/kg/day) found to reduce food intake in overweight, *ad libitum*-fed neutered cats [6], not evoking signs of estrus in ovariectomized cats [5], [31]. To our knowledge, the oral bioavailability of E2 in cats has not been reported. Nonetheless, it was expected to be low. Bioavailabilities of 4.3 and 10% are reported in rats and humans, respectively [32]. The decrement in food intake from the E2 dosage used to evaluate parameters of glucose homeostasis was within ranges observed in the dose response trial when oral dosage was 1 and 2 μg/kg/day (Fig 2).

Although food intake was decreased by E2, there was no significant effect on body weight (Table 3). Substantial weight loss was not a desired outcome because it could have confounded interpretation of the results. Specifically, overweight body condition in cats is known to affect glucose tolerance and indices of insulin sensitivity [33], [34].

The mechanism by which E2 treatment reduced food intake was not apparent. In humans and in rodents, E2 has been shown to affect the potency of peripheral feedback signals for meal size, specifically with respect to cholecystokinin (CCK) [35]. CCK is released from the small intestine in response to food intake and exerts a vagally-mediated satiating action which modulates meal size. Hence, a potentiating effect of E2 on CCK may have decreased food intake in the cats. Another speculation for the reduction in food intake is via a centrally mediated pathway. In ovariectomized rodent models, E2 overcomes arcuate leptin resistance, increases the secretion of anorectic neuropeptides (α-melanocyte-stimulating hormone), and decreases the secretion of orexigenic neuropeptides (neuropeptide Y and Agouti-related peptide) [36]. While this pathway has not been elucidated in cats, estrogen target neurons have been identified [37] and may be mediators of E2’s anorectic function.

We were not expecting the magnitude of variation in food intake between subjects with E2 treatment, particularly with respect to body weight (Fig 4). Although E2 did have an effect on food intake in all cats, it would appear that the obese versus the overweight cats were more refractory to the appetite suppressive effects. In this trial, one fixed dose of E2 was used for each subject. It is possible that the pharmacokinetics of exogenous E2 are such that it partitions into peripheral fat deposits and thus a higher dose is required in more obese individuals. Since adiposity does appear to affect food intake, a future direction is to evaluate indices of glucose homeostasis with stratified groups of cats according to body condition. The variability in food intake with respect to body weight also may have been a reflection of decreased E2 sensitivity in the obese cats. Individual differences in sensitivity of food intake to the residual estradiol after neutering might account for the large variation in body condition observed among cats post-neutering.

### E2 Effects on Glucose Homeostasis

Contrary to our hypothesis, glucose tolerance was not improved with E2 treatment as indicated from the IVGTT variables, clearance half-time, rate of disappearance (k), and plasma glucose AUC (Table 4). The early-phase insulin response (AUC_0–15 min_) also was not affected by the treatment. In contrast, an unexpected and important finding was the late-phase insulin response (AUC_60–120min_) being reduced with E2 treatment compared to the control (Fig 6).

### E2 Effects on Insulin Sensitivity

An increase in the late-phase insulin response of the IVGTT (AUC_60–120_) is observed when cats gain body weight in excess of ideal [16]. In accord with this, the AUC_60–120_ plasma insulin observations during the Vh treatments were positively correlated with body weight, which greatly differed among cats. Reduction in insulin sensitivity with weight gain in the non-diabetic is believed to account for an increased late-phase insulin response [38]. The present IVITT results support this belief.

Insulin sensitivity was measured as the fractional rate of glucose disappearance from plasma 15 minutes after IV insulin administration. This measure of insulin sensitivity was evaluated in lieu of other indicators because it directly measures a response to exogenously administered insulin, as is done with the more invasive measures of insulin sensitivity, the euglycemic-hyperinsulinemic clamp (EHC) [39], [40], [41], [34], and some forms of the Bergman Minimal Model, frequently-sampled IVGTT [29], [42]. In humans, the insulin sensitivity indicated by the IVITT significantly correlates with insulin sensitivity indicated by the EHC, a reputed “gold standard” measure [19], [20]. A similar validation of the IVITT insulin sensitivity index in cats is lacking. However, the presently determined k_*ivitt*_ values correlated well with previously reported “measures” of insulin sensitivity in cats (Table 5). Further, in the present study, insulin sensitivity indexes, *k*_ivitt_, decreased with increasing body weight. The heaviest cats were the least insulin sensitive and the lightest cats were the most insulin sensitive as has been previously reported using different measures of insulin sensitivity [18], [42], [34], [16].

Unexpectedly, the insulin sensitivity index (k_ivitt_) during E2 treatment was not significantly different from that during Vh treatment. This finding would appear to contrast with that seen in humans and in rodent models that lack endogenous E2 secretion (i.e. post-menopausal or ovariectomized). In these other species, E2 enhances insulin sensitivity, ameliorates insulin resistance, and has a protective effect against the development of type 2 DM [11]. It is possible that a greater number of animals and\or a more sensitive measure of insulin sensitivity may have shown a significant effect of E2 at the dose presently used. A post-hoc analysis of our observations on k_ivitt_ indicated that the experimental design was of sufficient power (β = 0.8) to detect a 25% difference in k_ivitt_ with treatment (α = 0.05).

However, a high or parenteral dosage of E2 may be required to produce an effect on insulin sensitivity in neutered cats. The oral E2 dosage used did not significantly increase systemic venous E2 concentration. Physiological effects of exogenous E2 appear to vary with dosage. While not reporting on food intake, Michael and Scott [31] showed that by increasing subcutaneous E2 dosage, the latency between E2 administration and observation of estrus in cats decreases to a minimum. Cave et al. [6] showed that the lowest effective subcutaneous dose of E2 to reduce food intake was not sufficient to cause vaginal cytological changes consistent with estrus nor obvious estrus behavior. Additional studies are required to evaluate the effects of route of administration and dosage of E2, within physiological bounds, on insulin sensitivity. When conducting such studies, variables not controlled in the present study should be considered. Research in rodents indicates effects of exogenous E2 may vary with the age of neutering, time of E2 initiation following neutering, and duration of E2 dosing after neutering [43].

Our finding of a decreased late-phase insulin response with no substantive effect on indices of glucose tolerance might be explained by an E2-mediated enhanced glucose effectiveness. Glucose effectiveness, which refers to the ability of glucose to enhance its own uptake and suppress endogenous production [43], was not evaluated in the present study. Additionally, the decreased insulin response with E2 treatment could have reflected reduced hepatic glucose production. Orally administered E2 can increase hepatic insulin sensitivity and insulin suppression of hepatic glucose production with no observable peripheral effect [44].

### E2 effects on Physical Activity

In this study, there was no significant effect of orally administered E2 on physical activity (Table 4). Testing for an effect on activity may have been confounded by between-cat differences in body condition. Physical activity of the leanest cats were most affected by E2 whereas the heaviest were least affected. We propose that a significant correlation between activity and body fat may have been detected if the cats had a uniform distribution in body fat.

In this study, physical activity was measured but not in lieu of energy expenditure. In ovariectomized mice, oral E2 administration decreases adiposity by increasing energy expenditure [42]. This effect of E2 is not known in cats. Here, we elected to evaluate physical activity by allowing daily social interaction with the opportunity for exercise. Decreased physical activity is a major risk factor in the development of feline DM [3] and E2 treatment reputedly increases physical activity in rodent models [12]. Measurement of energy expenditure would be useful for evaluating effects of E2 on energy homeostasis in cats and is therefore a worthy subject of future study.

## Conclusions

Exogenous E2 administered orally at a dosage of 1 μg/kg/day moderately reduced *ad libitum* food intake in overweight neutered male cats. The dosage was not supraphysiological nor emulating of the pre-gonadectomy condition as indicated from plasma E2 concentrations. The late-phase insulin response of the glucose tolerance test was reduced by approximately 1/3 with E2 treatment, and in accord with previous reports, the late-phase insulin response increased with body weight. Insulin sensitivity decreased with increasing body weight, also in accord with previous reports, but unlike in rodent models, insulin sensitivity was not significantly changed by E2 treatment. The E2 treatment had no significant effects on glucose tolerance and physical activity. We propose that oral E2 at the dose used may affect secretion or removal of insulin, and that it may enhance glucose disposal independent of insulin sensitivity. We suggest additional work in this feline model to determine if E2 increases glucose effectiveness and if increasing E2 dosage within a physiological range will increase insulin sensitivity, glucose tolerance, and physical activity as observed in other species.
